# A Prospective Study to Evaluate the Management of Sub-trochanteric Femur Fractures with Long Proximal Femoral Nail

**DOI:** 10.5704/MOJ.1711.014

**Published:** 2017-11

**Authors:** M Kumar, V Akshat, A Kanwariya, M Gandhi

**Affiliations:** Department of Orthopaedics, Dr SN Medical College and Hospital, Jodhpur, India; ^*^Department of Orthopaedics, Jhalawar Hospital and Medical College, Jhalawar, India; ^**^Department of Anaesthesia, Dr SN Medical College and Hospital, Jodhpur, India; ^***^Department of Paediatric Dentistry, Government Dental College, Jaipur, India

**Keywords:** sub-trochanteric fractures, Seinsheimer classification, long Proximal Femoral Nail

## Abstract

**Introduction:** Sub-trochanteric fractures of the femur remains one of the most challenging fractures faced by orthopaedic surgeons. This study was done to analyse the management and complications of sub-trochanteric fractures using long proximal femoral nail (PFN).

**Materials and Methods:** This was a prospective study of 50 patients with sub-trochanteric fractures of femur who were treated with long PFN at a tertiary care center from July 2012 to June 2016. The fractures were classified according to Seinsheimer classification. All patients were assessed functionally by Harris Hip Score.

**Results:** Average duration of union was 17.08 weeks (range 13 to 32 weeks), union was achieved in 92% cases. Closed reduction was achieved in 68% cases and open reduction was required in 32% cases. Various intraoperative complications were seen in 12% and delayed complications in 26% of cases. Good anatomical results were achieved in 86% of cases and 14% were fair. As per Harris Hip score, excellent results were noted in 28% cases, good in 56% cases and fair in 16% cases.

**Conclusion:** The long PFN is a reliable implant for subtrochanteric femur fractures, with high rate of bone union and minimal soft tissue damage. Intramedullary fixation has biological and biomechanical advantages, but the surgery is technically demanding.

## Introduction

Sub-trochanteric fractures are femoral fractures that involve the lesser trochanter and extend distally up to 5cm^1^. These fractures account for 10% to 34% of all hip fractures^2^. The sub-trochanteric region is usually exposed to high stresses during routine activities. Axial loading forces through the hip joint create a large moment arm, with significant lateral tensile stresses and medial compressive loads. In addition to the bending forces, muscle forces at the hip also create torsional effects that lead to significant rotational shear forces. In the sub-trochanteric region thickness of cortical bone is more and vascularity is less which produce healing disturbances.

High compressive and tensile forces of muscles separate the fracture fragments and cause instability of the fracture. Hence this fracture is difficult to manage and is associated with many complications including mal-union, delayed union, non-union and implant failure^3^. Due to these anatomical features conservative treatment is not preferred, and if there are no absolute contra indications and the patient can tolerate surgery, surgery is the treatment of choice^4^. The goal of operative treatment is restoration of normal length, anatomical alignment and angulation to restore adequate tension to the abductors. Early mobilization and weight bearing are possible with advances in implants and fixation technology. The two primary options for treatment of subtrochanteric fractures are intramedullary fixation and extramedullary fixation^5^.

Extramedullary implants including condylar blades plates and proximal femoral locking plates have been used to treat sub-trochanteric fractures, but they were associated with complications of high rate of reduction loss, fixation failure and the need for reoperation^6^. Compared with extramedullary implants, intra-medullary implants have several biomechanical advantages with benefits, including less soft tissue dissection, dynamic locking, ease of insertion, potentially less blood loss, restoration of the mechanical axis and, most importantly, allowance for immediate weight bearing after fixation^7^. There have few studies to compare results of these two modalities. The purpose of our study was to evaluate the results, complications and functional outcomes of long PFN in the management of subtrochanteric femur fractures.

## Materials and Methods

This prospective study was conducted on 50 patients with sub-trochanteric fractures of femur who were treated with long PFN in a tertiary care center from July 2012 to June 2016. Permission was obtained from the ethical committee in accordance with the Helsinki Declaration before starting the research. Informed consent was obtained from all the patients.

Pathological fractures, fractures in patients <18 years, old neglected fractures, peri-prosthetic and open fractures were excluded from the study. The fractures were classified according to Seinshemier classification. Appropriate length and diameter of long PFN, with distal diameter of 9, 10, 11, 12mm and proximal diameter of 14mm were used. Proximal locking was done using de-rotation screw of 6.5mm and distal lag screw of 8mm and distal locking with self-tapping 4.9mm cortical screws, one in static mode and the other in dynamic mode, allowing 5mm dynamization. The nails used were universal with 6^0^ of medio-lateral valgus angulation and neck shaft angle of 135^0^. End cap was not used.

**Fig. 1:**
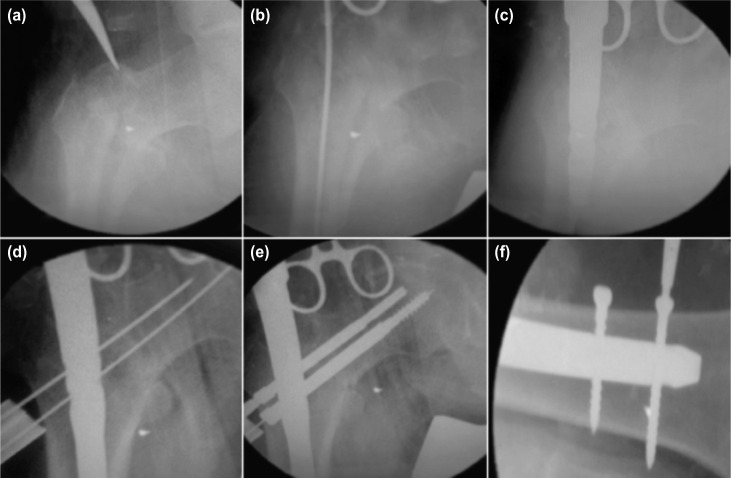
(a) Determination of entry point, (b) Insertion of guide wire (c) Insertion of long PFN, (d) Insertion of guide wire for compression and anti-rotation screw, (e) Insertion of compression screw and anti-rotation screw and (f) distal locking (static and dynamic mode).

After preoperative radiographic assessment and planning, the patient was prepared for surgery. The patient was placed in supine position on fracture table and the fracture was reduced by applying traction in external rotation and 20^0^ abduction (to correct varus deformity) and finally the limb was internally rotated up to neutral position and adducted. Preoperative prophylactic antibiotic was administered. The tip of greater trochanter was identified and a 5cm longitudinal incision was made proximal to it. Fascia lata and gluteus medius were incised in line with skin incision. The tip of the greater trochanter was exposed. Entry was made from tip or slightly lateral to the tip of greater trochanter on antero-posterior (AP) view in C-arm and in the centre of the medullary cavity in the lateral view (Fig. 1a). Medullary canal was entered with a curved bone awl and guide wire was inserted into the medullary canal (Fig. 1b). Using a cannulated conical reamer, the proximal femur was reamed for a distance of about 7cm. After confirming satisfactory fracture reduction, an appropriate size nail was inserted as far as possible into the femoral opening until the hole for 8mm screw was at the level of the inferior margin of the femoral neck (Fig. 1c).

Jiang *et al*^8^ recommended that lag screw of long PFN should be placed in the lower part of the neck close to the femoral calcar with the screw tip reaching the subchondral bone 5mm below the articular cartilage in the AP view. In the lateral view it should be placed in the centre of the femoral neck, thereby the lag screw would be definitely placed in the area of best bone quality. A 2.8mm guide wire was inserted through the drill sleeve after a stab incision, 5mm deeper than the planned screw size. It’s final position was kept in the lower half of the neck in the AP view and in the centre of the neck in the lateral view. For anti-rotation screw a second wire was inserted in the similar way above the first one (Fig. 1d). The anti-rotation screw was inserted first to prevent possible rotation of the medial fragment and to reduce chances of varus angulation when inserting the compression screw. The length of anti-rotation screw was measured and 5mm was deducted from it. Drilling was done over the guide wire with 6.5mm drill bit up to the length of anti-rotation screw previously measured. Tapping was not done as neck screw was self-tapping. Compression screw was placed in similar manner by drilling with 8mm reamer (Fig. 1e). Distal locking was performed with two cortical screws by drilling with a 4mm drill bit and position confirmed with image intensifier (Fig. 1f). Open reduction was performed in cases in which satisfactory reduction was unsuccessful. After fixation, closure was done in layers. Suction drain was used in cases of open reduction similar to Verley *et al*^9^. IV antibiotics were continued for three days post-operatively which was prolonged to seven days in cases where open reduction was performed, followed by oral antibiotics for five days. Analgesics were given as required, and the procedures are illustrated in Fig.1 a-f.

**Fig. 2:**
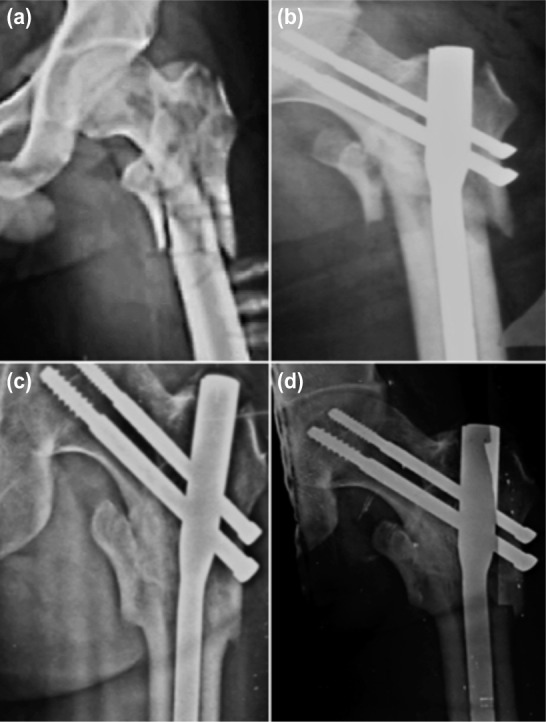
AP radiographs (a) Pre-op, (b) Post-op, (c) three month follow-up and (d) six month follow-up.

Static quadriceps exercises and knee mobilization were started in the immediate postoperative period. On the 4th-5th postoperative day, depending on the patients’ pain tolerance, they were made to stand up with assistance, and gradually over the following two to three days they were allowed non-weight bearing walking with frame. Patients were discharged on the 10th day after suture removal. Partial weight bearing was started at four weeks in all patients and full weight bearing at 12 weeks. All patients were followed up at 4 weeks, 12 weeks and thereafter at 6 weekly intervals, till fracture union was noted, then at 6 months, 9 months and one year. At each visit, patient was assessed clinically for hip and knee function, walking ability, fracture union, deformity and shortening. During follow-up AP and lateral radiographs of pelvis with both hips were obtained (Fig. 2 a-d). Assessment of functional results was based on Harris Hip Scoring System.

## Results

**Fig. 3:**
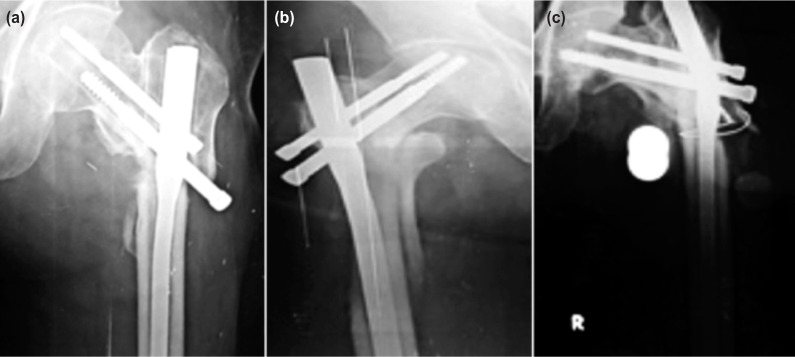
(a) Z-effect, (b) Fracture of lateral cortex and (c) Implant failure.

In our study, all the patients were operated at an average interval of 14.2 days from the day of trauma. They were between 31 to 60 years of age group with mean age of 48.72 years. There were 38 males and 12 females. The majority of fractures were right-sided and road traffic accidents accounted for most of the cases, followed by fall from height. As per Seinshemier classification there were 28 cases of Type II, 12 cases of Type III, 4 cases of Type IV and 6 cases of Type V. The most commonly used compression screw was size 100mm and the anti-rotation screw was 85mm. Average union duration was 17.08 weeks (range 13 to 32 weeks), 32 cases showed union at four months, eight cases at five months and six cases at six months duration. Mean duration of surgery was 60.4 minutes (range 35-105). Duration of surgery was longer in the initially operated cases and in managing sub-trochanteric fracture Type IIb. Average duration of radiation exposure was 115 seconds. Radiation exposure was high in initial cases due to lack of experience and more in closed reduction cases and for comminuted fracture with difficult reduction. Average amount of blood loss was 164.6ml (range 70-280ml) measured by mop count (each fully soaked mop containing 50ml blood). There was greater blood loss in open reduction cases but with meticulous dissection and by preventing damage to the perforator we reduced blood loss significantly. Intraoperative and delayed complications are summed up in Table I and II and radiographs (Fig. 3 a-c.) Anatomical results were assessed by presence or absence of shortening, varus deformities and range of movements in hip and knee joints. Good results were noted in 86% of cases and 14% fair results (Table III). Functional results assessed by Harris Hip Scoring System gave excellent results in 14 (28%) cases, good in 28 (56%) cases and fair in 8 (16%) cases (Table III).

**Table I:** Intra Operative Complications

Complications	Frequency	Percentage (%)
Failure to achieve closed reduction	16	32
Fracture of lateral cortex	2	4
Failure to put anti-rotation screw	2	4
Guide wire breakage	2	4

**Table II:** Delayed Complications

Complications	No of cases	Percentage (%)
Hip joint stiffness	3	6
Knee joint stiffness	1	2
Delayed union	2	4
Implant failure	2	4
Z-effect (cut of lag screw)	4	8
Varus angulation	4	8
Superficial infection	2	4
Reverse Z-effect	0	0

**Table III:** Anatomical Results

Anatomical results	Frequency	Percentage (%)
Restriction of hip ROM	3	6
Restriction of knee ROM	1	2
Shortening >1 cm	0	0
Varus deformity	4	8

## Discussion

Sub-trochanteric fractures are usually the result of high-energy trauma and often subjected to significant displacement with difficulty in closed reduction with traction. The high incidence of delayed union, malunion and non-union of fractures has left conservative treatment abolished in modern trauma care^10^. Extra medullary fixation with plating has the potential disadvantages of extensive surgical exposure, severe soft tissue damage and blood loss, thus leading to problems of fracture union and implant failure. In addition, the eccentric plating is prone to fatigue breakage due to mechanical load-sharing effect. In a minimally open approach, intramedullary nailing is closely linked to “biological internal fixation”, in addition to its mechanical benefits over plate fixation. Intramedullary fixation allows the surgeon to minimize soft tissue dissection, thereby reducing surgical trauma, blood loss, infection, and wound complications^11^. Good reduction with minimal dissection, use of appropriate nail length and proper positioning of the nail and screws are necessary to avoid failure or revision. The abundant muscles around the sub trochanteric region usually cause significant displacement of the fractured fragments, leading to great difficulties in close reduction under traction. Sometimes open reduction through a small incision at the fracture site is inevitable.

The average union rate reported in the literature was from 85-100%. Delayed union and non-union, which are common complications of these fractures, have been reported to be 1-10% in various studies^4,8,12-15^. In our study union rate in six months was 92%. In 4% cases of Type IIb, union was delayed due to inadequate reduction of fracture, for which dynamization was done with fracture union on follow-up in both cases. Two cases of non-union due to implant breakage at four and six months were treated with exchange nailing and the fractures united three months thereafter.

Open reduction was performed in 32% cases out of which 24% were Type IIb fractures, 4% cases each of Type IIIb and Type V fractures. Difficulty in reduction was probably due to the integrity of lesser trochanter and muscle pull which produce characteristic displacement of flexion, abduction and external rotation of proximal fragment in Types IIb and IIIb and due to severe comminution at fracture site in Type V fractures. In the study by Jiang *et al*^8^, open reduction was done in 34% cases and no intraoperative fracture of bone or breakage of implant were noted. Zhou *et al*^4^ open reduced 9% of the cases in their study. Iatrogenic fracture of lateral cortex of proximal fragment was seen in 4% cases, plausibly due to wrong entry point in initially operated cases. It’s incidence decreased with increased experience. In 4% cases failure to place both proximal screws together was seen, hence proximal locking was left with only neck screw (compression screw) thus decreasing stability of construct. While drilling over guide wire for applying compression screw it broke inside the neck in 4% cases; later on it was removed and compression screw placed successfully. Review of literature revealed similar intraoperative complications^8,12,14^. In our study superficial wound infection was seen in 4% cases, in which open reduction was performed, and infection resolved with intravenous antibiotics continued for three weeks. Yadav *et al*^14^ in their study reported infection in 6.67% cases. Wound complication rate in our study was less compared to other studies because of the longer administration of antibiotics in cases of open reduction.

Various mechanical complications associated with PFN were reported by many authors including Z-effect (cut-out of screw), reverse Z-effect, implant failure and failure of fixation requiring re-operation. Werner *et al*^16^ were the first to introduce the term Z-effect, detected in 7% of their cases. The Z-effect phenomenon was described as a characteristic lateral migration of the inferior screws, varus collapse of the fracture and perforation of the femoral head by superior screw during the postoperative weight bearing period. They proposed that fixation of the fracture at a cervico-diaphysial angle of <125^0^ was a predisposing factor for the Z-effect and reverse Z-effect, as well as for cut-out of screws. Strauss *et al*^17^ have reproduced the migration of the cephalic screws from the intra medullary nail in the laboratory with the aid of a polyurethane model and observed that when compressive forces on the femoral head and bone density were greater than those on the femoral neck, inferior screw migrated laterally.

The reverse Z-effect as described by Boldin *et al*^13^ involves the lateral migration of the superior screw accompanied by the medial migration of the inferior screw, which required early removal. Simmermacher *et al*^18^ in their study had implant failure and cut-out of screw in 0.6% cases each. Rate of cut-out of screw and implant failure in literature varies from 1-11% and 1-7% respectively. Average rate of Z-effect in various literature was 3-6% and that of reverse Z-effect was 2-4%^4,8,12-15^. In the study by Yadav *et al*^14^ shortening >1cm and varus deformity were seen in 4% cases. Rate of reoperation for non-union or implant failure or Z-effect has been reported from 9% to as high as 29%. In our study, Z-effect was seen in 8% cases and no cases of reverse Z-effect and femoral fracture below the tip of PFN were seen. Re-operation due to implant breakage and Z-effect in our study was in 12% of cases.

Excellent results were noted in 14 (28%) cases, good in 26 (56%) cases and fair in 8 (16%) cases, as per Harris Hip Score in our study. Other studies^4,14,15^ in which Harris Hip Score was used also showed similar outcomes as our study. Favourable results seen in 84% of patients managed by long PFN may be explained, on the basis as stated by Leung *et al*, and Radford *et al*^11^ that intramedullary nailing by allowing a minimally open approach which is closely linked to “biological internal fixation”, in addition to its mechanical benefits over plate fixation also allows the surgeon to minimize soft tissue dissection, thereby reducing surgical trauma, blood loss, infection and wound complications.

## Conclusion

Osteosynthesis with the proximal femoral nail offers the advantages of high rotational stability of the head-neck fragment, compression at fracture site and is biomechanically sound as it is an intramedullary device, thus leading to minimal soft tissue damage and high rate of bone union. Most of the complications of proximal femoral nailing are related to the surgeon and instruments, which can be reduced by proper patient selection and good preoperative planning. Gradual learning and patience are needed to make this method truly minimally invasive.
